# Biosystems Design to Accelerate C_3_-to-CAM Progression

**DOI:** 10.34133/2020/3686791

**Published:** 2020-10-10

**Authors:** Guoliang Yuan, Md. Mahmudul Hassan, Degao Liu, Sung Don Lim, Won Cheol Yim, John C. Cushman, Kasey Markel, Patrick M. Shih, Haiwei Lu, David J. Weston, Jin-Gui Chen, Timothy J. Tschaplinski, Gerald A. Tuskan, Xiaohan Yang

**Affiliations:** ^1^Biosciences Division, Oak Ridge National Laboratory, Oak Ridge, TN 37831, USA; ^2^The Center for Bioenergy Innovation, Oak Ridge National Laboratory, Oak Ridge, TN 37831, USA; ^3^Department of Genetics and Plant Breeding, Patuakhali Science and Technology University, Dumki, Patuakhali 8602, Bangladesh; ^4^Department of Genetics, Cell Biology and Development, Center for Precision Plant Genomics, and Center for Genome Engineering, University of Minnesota, Saint Paul, MN 55108, USA; ^5^Department of Applied Plant Sciences, Kangwon National University, Chuncheon 24341, Republic of Korea; ^6^Department of Biochemistry and Molecular Biology, University of Nevada, Reno, NV, USA; ^7^Department of Plant Biology, University of California, Davis, Davis, CA, USA; ^8^Feedstocks Division, Joint BioEnergy Institute, Emeryville, CA, USA

## Abstract

Global demand for food and bioenergy production has increased rapidly, while the area of arable land has been declining for decades due to damage caused by erosion, pollution, sea level rise, urban development, soil salinization, and water scarcity driven by global climate change. In order to overcome this conflict, there is an urgent need to adapt conventional agriculture to water-limited and hotter conditions with plant crop systems that display higher water-use efficiency (WUE). Crassulacean acid metabolism (CAM) species have substantially higher WUE than species performing C_3_ or C_4_ photosynthesis. CAM plants are derived from C_3_ photosynthesis ancestors. However, it is extremely unlikely that the C_3_ or C_4_ crop plants would evolve rapidly into CAM photosynthesis without human intervention. Currently, there is growing interest in improving WUE through transferring CAM into C_3_ crops. However, engineering a major metabolic plant pathway, like CAM, is challenging and requires a comprehensive deep understanding of the enzymatic reactions and regulatory networks in both C_3_ and CAM photosynthesis, as well as overcoming physiometabolic limitations such as diurnal stomatal regulation. Recent advances in CAM evolutionary genomics research, genome editing, and synthetic biology have increased the likelihood of successful acceleration of C_3_-to-CAM progression. Here, we first summarize the systems biology-level understanding of the molecular processes in the CAM pathway. Then, we review the principles of CAM engineering in an evolutionary context. Lastly, we discuss the technical approaches to accelerate the C_3_-to-CAM transition in plants using synthetic biology toolboxes.

## 1. Introduction

The global population has quadrupled over the past 100 years and will continue to increase in the 21^st^ century [1]. To feed the growing population, crop production must increase, either by expanding the amount of agricultural land for growing crops or by increasing crop yields on existing agricultural lands. Simultaneously, ongoing and projected climate changes are (1) affecting many sectors important to society, including human health, agricultural sustainability, water supply, energy security, and food supply and (2) becoming increasingly disruptive in the coming decades [2–4]. These opposing trends are threatening our global food and energy security [5]. To meet this challenge, various approaches have been explored to increase the productivity of agricultural crops [6–9]. Among them, one of the most direct approaches is engineering crassulacean acid metabolism (CAM) into C_3_ crops to enhance water-use efficiency (WUE) in plants [9] thereby allowing such crops to be grown on marginal lands with reduced fresh water inputs.

To adapt to various environments on Earth, plant species have evolved several photosynthetic chemistries—C_3_, C_4_, and CAM photosynthesis [10]. The way plants fix atmospheric CO_2_ is the key to distinguish different photosynthesis. C_3_ photosynthesis is a one-stage process that produces a three-carbon compound (3-phosphoglyceric acid) via the Calvin-Benson-Bassham (CBB) cycle, while C_4_ photosynthesis and CAM photosynthesis are two-stage processes, with the fisrt stage fixing CO_2_ into a series of four-carbon compounds from oxaloacetate to malate, followed by the secondary stage, where four-carbon compounds are decarboxylated, releasing CO_2_ to be refixed via the CBB cycle. In C_4_ plants, photosynthesis is separated spatially (mesophyll and bundle sheath cells), whereas in CAM photosynthesis CO_2_ fixation is separated temporally (day and night). In CAM plants, stomata close during part or all of the day to reduce water loss, and the CO_2_ is released from the malate generated during the first CO_2_-fixing stage, resulting in enhanced plant WUE in comparison with C_3_ or C_4_ plants. WUE is the crop’s ability to assimilate a unit of carbon per unit of water consumed [11]. However, gas exchange in the leaf to obtain CO_2_ inevitably results in water loss. The CAM solution to this problem is to open the stomata at night and fix carbon into malic acid, then close the stomata during the heat of the day, and release the stored CO_2_ to the CBB cycle, maximizing WUE. Typically, CAM species have very high WUE, at least six- and three-fold greater than that of C_3_ and C_4_ plants, respectively [12].

Fresh water is the most critical resource of sustainable agriculture, and approximately 42% of the land area on Earth is classified as dryland [13, 14], where precipitation is inadequate for major conventionally grown C_3_ or C_4_ crops. Bioengineering CAM into C_3_ plants is a potential solution to these challenges. However, engineering a major metabolic pathway like CAM is not a trivial task. Not only does it require a deep understanding of the metabolic and regulatory pathways during CAM photosynthesis, but also it requires precise regulation of the enzymatic activities, intracellular transporters, and stomatal conductance [9, 15, 16].

CAM species have been increasingly considered important climate-resilient species in the world and are a crucial driving force of ecosystem function in arid areas [17]. Recently, important achievements were made in CAM plant genomics research, significantly increasing our knowledge on the molecular mechanisms underlying CAM photosynthesis [17–20]. However, the application of this basic knowledge to CAM engineering is still limited due to technical challenges, including the lack of robust biosystems design capabilities for reconfiguring signaling and metabolic pathways in plants. Recently, biosystems design, integration of systems biology, and synthetic biology based on genome editing have emerged as innovative approaches for genetic improvement of complex biological systems in plants, microbes, and animals [21]. And as such, opportunities for revolutionizing agriculture with synthetic biology are emerging [22].

This review is intended to inspire the utilization of a biosystems design approach to accelerate C_3_-to-CAM progression. First, we provide a summary of the molecular mechanisms underpinning CAM photosynthesis based on systems biology research. Second, we discuss the principles of CAM engineering in an evolutionary context. Lastly, we integrate the capabilities of gene editing and synthetic biology for CAM engineering, with a focus on building a CAM-on-demand system to increase plant resistance to episodic or seasonal drought stress.

## 2. A Systems Biology-Level Understanding of CAM Photosynthesis

**Figure 1:**
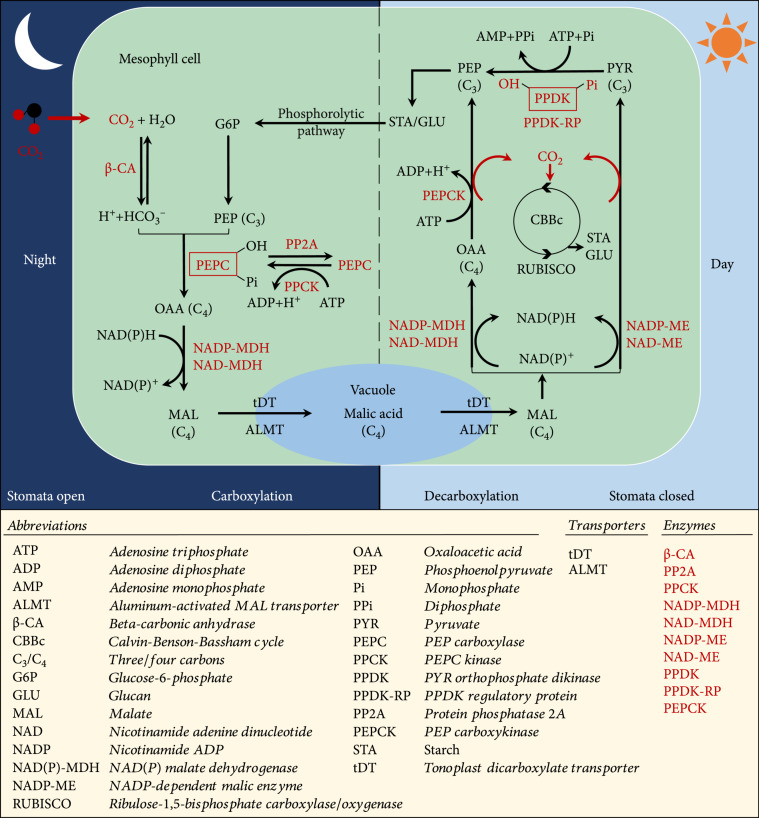
A simplified view of the crassulacean acid metabolism (CAM) photosynthetic pathway including key enzymes, regulatory proteins, and transporters.

The exploration of the molecular mechanisms of CAM is critical for CAM engineering in C_3_ plant species. CAM features four core functional modules: (1) a carboxylation module to fix CO_2_ and accumulate malic acid in the vacuole during the nighttime, (2) a decarboxylation module to release CO_2_ from malic acid during the daytime for refixation mediated by ribulose-1,5-bisphosphate carboxylase/oxygenase (Rubisco) [9, 23] (Figure 1), (3) a stomatal control module to open stomata during the night and close them during the day, and (4) an anatomical module to increase the succulence of the leaf tissue [9]. A distinctive feature of CAM plants is that the stomata in the leaves remain closed during most or all of the daytime but open during the nighttime to take up CO_2_, reducing water loss and correspondingly increasing WUE due to the lower evapotranspiration rates at night. Over the past ten years, genes in these functional modules (Table 1) have been identified using systems biology approaches, which involved multiomics (e.g., genomics, transcriptomics, metabolomics, and proteomics), metabolic modeling, and molecular genetic technologies such as RNA interference (RNAi) and gene editing mediated by clustered regularly interspaced short palindromic repeats (CRISPR)/CRISPR-associated (Cas) systems.

**Table 1:** List of known genes within the functional CAM modules.

Protein name	Gene locus	Definition (subcellular location)	Species	Reference
Carboxylation module				
*β*-CA	Kaladp0018s0289	*β*-Type carbonic anhydrase	*K. fedtschenkoi*	[20]
*β*-CA2	Mcr010929t1	*β*-Type carbonic anhydrase 2 (cytosol)	*M. crystallinum*	[30]
PEPC1	Kaladp0095s0055	Phosphoenolpyruvate carboxylase 1	*K. fedtschenkoi*	[20]
PEPC1	Mcr000915t1	Phosphoenolpyruvate carboxylase 1 (cytosol)	*M. crystallinum*	[30]
PEPC1	Kalax.0018s0056.1	Phosphoenolpyruvate carboxylase 1	*K. laxiflora*	[35]
	Kalax.0021s0061.1			
PEPC2	Kaladp0048s0578	Phosphoenolpyruvate carboxylase 2	*K. fedtschenkoi*	[20]
PPCK	Kaladp0037s0517	PEPC kinase	*K. fedtschenkoi*	[20]
PPCK1	Mcr011042t1	PEPC kinase 1 (cytosol)	*M. crystallinum*	[30]
NAD-MDH	Kaladp0022s0111	NAD-malate dehydrogenase	*K. fedtschenkoi*	[20]
NAD-MDH1	Mcr009416t1	NAD-malate dehydrogenase 1 (cytosol)	*M. crystallinum*	[30]
NAD-MDH2	Mcr008974t1	NAD-malate dehydrogenase 2 (mitochondria)	*M. crystallinum*	[30]
NADP-MDH1	Mcr006398t1	NADP-malate dehydrogenase 1 (chloroplast)	*M. crystallinum*	[30]
ALMT6	Kaladp0062s0038	Tonoplast aluminum-activated malate transporter 6	*K. fedtschenkoi*	[20]
ALMT4		Tonoplast aluminum-activated malate transporter 4 (tonoplast membrane)	*M. crystallinum*	Lim et al., unpublished data
tDT		Tonoplast dicarboxylate transporter	*Agave*	[37]
tDT		Tonoplast dicarboxylate transporter (tonoplast membrane)	*M. crystallinum*	Lim et al., unpublished data

Decarboxylation module				
NAD-ME		NAD-dependent malic enzyme	*K. fedtschenkoi*	[20]
NAD-ME1	Mcr021367t1	NAD-dependent malic enzyme 1, alpha subunit (mitochondria)	*M. crystallinum*	[30]
NAD-ME2	Mcr003267t1	NAD-dependent malic enzyme 2, beta subunit (mitochondria)	*M. crystallinum*	[30]
NADP-ME	Kaladp0092s0166	NADP-dependent malic enzyme	*K. fedtschenkoi*	[20]
NADP-ME1	Mcr003238t1	NADP-dependent malic enzyme 1 (cytosol)	*M. crystallinum*	[30]
NADP-ME2	Mcr002920t1	NADP-dependent malic enzyme 2 (chloroplast)	*M. crystallinum*	[30]
PPDK	Mcr000976t1	Pyruvate, orthophosphate dikinase (chloroplast)	*M. crystallinum*	[30]
PPDK-RP	Kaladp0010s0106	Pyruvate, orthophosphate dikinase-regulatory protein	*K. fedtschenkoi*	[20]
PPDK-RP	Mcr007074t1	Pyruvate, orthophosphate dikinase (chloroplast)	*M. crystallinum*	[30]
PEPCK		Phosphoenolpyruvate carboxykinase	*K. fedtschenkoi*	[20]
PPCK1	AF162661	Phosphoenolpyruvate carboxykinase	*K. fedtschenkoi*	[25]
PEPCK		Phosphoenolpyruvate carboxykinase (cytosol)	*M. crystallinum*	[30]

Stomatal regulation module				
PHOT2	Kaladp0033s0113	Blue light receptor phototropin 2	*K. fedtschenkoi*	[34]
AKT2		Arabidopsis shaker family K^+^ channels 2/3	*Agave*	[37]
PP1		Protein phosphatase 1	*K. pinnata*, *K. daigremontiana*	[33]
PM H^+^-ATPases		Plasma membrane H^+^-ATPase	*K. pinnata*, *K. daigremontiana*	[33]

Anatomical module				
VvCEB1		Basic helix-loop-helix transcription factor	*Vitis vinifera*	[59, 60]
PeXTH		The xyloglucan endotransglucosylase/hydrolase	*P. euphratica*	[57]
Circadian clock module				
CCA1		Circadian clock associated 1	*Agave*	[37]
TOC1		Timing of cab expression1	*Agave*	[37]
RVE1		Reveille 1	*Agave*	[37]

### 2.1. Genes in the CAM Carboxylation Module

After atmospheric CO_2_ enters the mesophyll cells, it is converted to HCO_3_^-^ by beta-carbonic anhydrase (*β*-CA), which is further, in combination with phosphoenolpyruvate (PEP), converted to oxaloacetate (OAA) by PEP carboxylase (PEPC) in the cytosol [24]. In most CAM plants, the reversible phosphorylation-dephosphorylation of PEPC mediated by PEPC kinase (PPCK) and possibly protein phosphatase 2A (PP2A) is understood to be under the control of the circadian clock (Figure 1) [25]. *PEPC1* and *PEPC2* are two most abundant PEPC transcripts in *Kalanchoe fedtschenkoi*. Two different patterns of convergent evolution are understood to be relevant to the carboxylation module. In the first pattern, the shift of PPCK expression from the light period to the dark period promoted the activation of PEPC1, as revealed in *K. fedtschenkoi* and *Ananas comosus* [20, 25]. In the second pattern, a single amino acid change from an arginine (R)/lysine (K)/histidine (H) to an aspartic acid (D) residue at the 509^th^ position counting from N-terminal occurred to keep PEPC2 active without being phosphorylated, as observed in CAM species *Phalaenopsis equestris* and *K. fedtschenkoi* [20]. Then, NAD(P)-malate dehydrogenase (NAD(P)-MDH) converts OAA to malate, which is transported into the vacuole by an aluminum-activated malate transporter (ALMT) or a tonoplast dicarboxylate transporter (tDT) (Figure 1) [26–29]. Recently, ectopic overexpression of each of the five individual carboxylation proteins (*β*-CA2, NAD-MDH1, NAD-MDH2, PEPC1, and PPCK1) from *Mesembryanthemum crystallinum*, which is a facultative CAM species, enhanced leaf growth, along with an increase in organic acid accumulation and stomatal conductance in *Arabidopsis thaliana* [30]. The increased plant size and biomass yield in the transgenic *Arabidopsis* plants might arise from the release of intracellular CO_2_, which reduced photorespiration and consequently promoted plant growth [30].

### 2.2. Genes in the CAM Decarboxylation Module

During the daytime, the malic acid is moved out of the vacuole and subsequently decarboxylated to release CO_2_ for Rubisco-mediated refixation in the chloroplast, generating carbohydrates through the CBB cycle (Figure 1). Two different likely species-dependent processes for malate decarboxylation occur according to whether the plants contain high levels of PEP carboxykinase (PEPCK) or NAD(P)-malic enzyme (NAD(P)-ME) (Figure 1). In the NAD(P)-ME-mediated decarboxylation process, malate is converted by NAD(P)-ME to pyruvate, along with the release of CO_2_ in the cytosol (or mitochondria/chloroplast), followed by subsequent conversion of pyruvate to PEP mediated by pyruvate orthophosphate dikinase (PPDK). In this process, the reversible phosphorylation-dephosphorylation of PPDK, catalyzed by the PPDK regulatory protein (PPDK-RP), results in activation-inactivation of PPDK in the light-dark cycle [31]. In the PEPCK-mediated decarboxylation process, NAD(P)-MDH converts malate to OAA, which is subsequently decarboxylated to PEP and CO_2_ by PEPCK (Figure 1). PEP is then metabolized into starch or other storage glucans and stored in plants during the day. The starch or other stored carbohydrates can be converted back to PEP via glycolysis to fuel subsequent carboxylation at night (Figure 1). In the same study mentioned above [30], ectopic overexpression of five decarboxylation proteins (NADP-ME1, NADP-ME2, NAD-ME1, NAD-ME2, and PPDK) from *M. crystallinum* also increased plant size, along with a decrease in stomatal conductance and accumulation of organic acid caused by *NADP-ME1* and *NADP-ME2* in *A. thaliana*.

### 2.3. Genes Affecting Stomatal Movement

The typical gas exchange pattern in CAM plants shows extensive interspecific, intraspecific, and intraindividual variation, which complicates the study of stomatal movement. Multiple factors, including blue light, leaf-air vapor pressure deficit (VPD), leaf water status, and intercellular CO_2_ concentration (C${}_{i}$), affect the regulation of a stomatal aperture [32]. Recently, protein phosphatase 1 (PP1) and plasma membrane (PM) H^+^-ATPase were shown to play crucial roles in the blue light-dependent stomatal opening in *K. daigremontiana* and *K. pinnata*, which are two obligate CAM species [33]. Furthermore, knocking out of blue phototropin 2 (KfePHOT2), a light receptor, reduced stomatal conductance and Rubisco-mediated CO_2_ fixation in the late afternoon when stomata are reopened and enhanced stomatal conductance and the nighttime CO_2_ fixation in the CAM species *K. fedtschenkoi* [34]. RNAi-mediated knockdown of the CAM PEPC isozyme (PEPC1) in *K. laxiflora* disrupts the dark period CO_2_ fixation and stomatal conductance and alters the temporal phasing of expression of genes controlling the movement of stomata, suggesting that inverse stomatal behavior is also likely to be dependent upon the activity of the primary carboxylation reaction [35]. Leaf water status usually acts on an ABA-dependent stomatal aperture in CAM plants [36–38]. C${}_{i}$ is a key driving force for CAM stomatal rhythm, which indicates the importance of metabolic control of stomatal movement in CAM plants [39, 40]. However, the key genes involved in leaf water status and C${}_{i}$ remain to be determined in CAM plants. Although the circadian oscillator can shape the rhythms of stomatal movement in CAM plants, it might not be as important as that in C_3_ plants [32]. Recently, numerous candidate genes were predicted to be involved in stomatal opening and closing in CAM plants [41]. More recently, over 200 *K. fedtschenkoi* genes were predicted to be relevant to stomatal movement [42]. Although it would be very challenging to engineer stomatal movement, there is precedence using small molecules to control stomata [43–45]. This could be used to provide proof-of-concept studies for CAM engineering.

### 2.4. Genes in the Anatomical Module

Besides the critical role of temporal gene expression in CAM plants, specific functional anatomical traits are thought to be associated with optimal CAM function [46–48]. Enlarged cells allow for a larger amount of organic acids to be stored in the vacuole during the nighttime [49] and also potentially enhance water uptake and remobilization in the chlorenchyma [50]. Densely packed mesophyll cells can reduce CO_2_ conductance ($g_{\text{m}}$) within the leaf and CO_2_ efflux from the leaf, increasing the capacity for performing CAM [46, 51, 52]. In typical CAM species, leaf thickness and cell size are increased whereas intracellular air space (IAS) and the length of mesophyll surface exposed to IAS per unit area ($L_{\text{mes}}/\text{area}$) are reduced in comparison with non-CAM plant species [47]. For example, leaf thickness as a measure of tissue succulence has been associated with the performance of CAM in the Crassulaceae [53], the Orchidaceae [54], and other CAM families [47]. A comparative analysis of phylogenetically unrelated C_3_+CAM and strong CAM species revealed that cell size was not related to CAM, reduced IAS and $L_{\text{mes}}/\text{area}$ were associated with CAM, and there was no difference in the proportion of IAS and $L_{\text{mes}}/\text{area}$ between strong and weak CAM species [46]. Also, a comparative analysis of multiple *Clusia* species ranging from C_3_ to CAM with intermediates showed that the proportion of CO_2_ uptake during the nighttime was significantly correlated with the size of palisade mesophyll cells [55]. However, in *Yucca gloriosa*, which is a C_3_+CAM hybrid species, leaf anatomy and CAM function were not well correlated, suggesting that CAM evolution can proceed initially through different combinations of multiple traits, and then, more favorable trait combinations are selected to form strong CAM species [56].

Several strategies have proven successful in increasing leaf and tissue succulence in C_3_ species with beneficial traits. For example, overexpression of the *Populus euphratica* xyloglucan endotransglucosylase/hydrolase gene (*PeXTH*) in tobacco decreased IAS within the palisade parenchyma, along with an increase in both leaf water content and cell packing, leading to improved salinity tolerance, presumably due to a reduction in the content of intercellular NaCl within leaf tissues [57]. Also, ectopic expression of a codon-optimized form (*Vv*CEB1_opt_) of the grape gene *VvCEB1*, which encodes a transcription factor in the basic helix-loop-helix (bHLH) family [58], increased organ and cell size, vegetative and reproductive biomass, and seed yield in *A. thaliana* [59]. Furthermore, overexpressing *VvCEB1* was shown to increase tissue succulence and decrease intercellular air space (IAS), leading to a leaf anatomy that could potentially optimize the performance of CAM [60]. In the *Vv*CEB1_opt_-overexpressing lines, the integrated and instantaneous WUE were increased, resulting in dramatically improved drought tolerance, along with enhanced salt tolerance due to a decrease in salinity uptake as well as a dilution of internal Na^+^ and Cl^-^ within the succulent leaves [60].

Besides the gene products involved in the above CAM modules, many other gene products are also implicated to function in CAM, such as starch phosphorylase, which is involved in the formation of PEP by glycolysis [61], and gene products involved in the regeneration of storage carbohydrates. Recently, at least 60 genes that are potentially involved in CAM evolution were identified in a comparative analysis of three obligatory CAM species (*K. fedtschenkoi*, *P. equestris*, and *A. comosus*) and some non-CAM plant species [20] or by comparison of nonphotosynthetic and photosynthetic tissues in *A. comosus* [41]. Among these genes predicted to be involved in CAM evolution, 54 genes displayed rewired diel patterns of gene expression and 6 genes showed protein sequence mutations [20]. The functional analysis of individual CAM-related genes by either overexpression [30], knockdown [25, 35, 39], or knockout [34] has laid a solid foundation for CAM biodesign. A functional CAM pathway is unlikely to result from single-gene engineering in C_3_ plants [30]. Clearly, the engineering of multiple genes related to CAM in a modular manner is necessary to recapitulate partially or fully functional CAM modules or pathways. To move forward, the future effort for engineering CAM in C_3_ plants should focus on the coordinated expression of the genes involved in carboxylation and decarboxylation in a manner as displayed by CAM species. Also, CAM engineering requires precise dynamic control of carbohydrate transportation, degradation, and storage to supply PEP, which is required for the PEPC-mediated carboxylation process during the nighttime.

## 3. The Progress in the Understanding of CAM Evolution

C_3_ photosynthesis is the predominant route that plants take in CO_2_ and produce carbohydrates, representing approximately 95% of the Earth’s plant biomass [62]. In contrast, C_4_ and CAM species, derived from C_3_ ancestors, account for about 3% and 6% of flowering plant species, respectively [63, 64]. Among the angiosperms (flowering plants), C_4_ photosynthesis has evolved independently at least 61 times in 19 families, and CAM has evolved independently in more than 400 genera across more than 38 families [15, 17, 65, 66]. Therefore, CAM photosynthesis and C_4_ photosynthesis are thought to be the result of convergent evolution from independent C_3_ plant lineages [15]. Among the 60 candidate genes underpinning the convergent evolution of CAM from diverse lineages of C_3_ plants, 90% showed rewiring of diel gene expression [20]. Interestingly, all of the enzymes in CAM seem to have homologs in C_3_ species [67, 68]. Shared biochemical properties suggest that the repeated, independent CAM and C_4_ evolution is due to the reorganization of coopted and modified ancient metabolic pathways [69]. The involved modifications can be initiated by mutation(s) and then accommodated under selection by genomic change as the adaptive phenotype evolves [70]. Indeed, C_4_ evolution is thought to require an enabling mutation to form an initial C_4_ cycle, followed by selection for loss of high expression of photorespiratory genes in a certain cell type [71]. However, an enabling mutation is hypothesized not to be required for the evolution of CAM [67].

**Figure 2:**
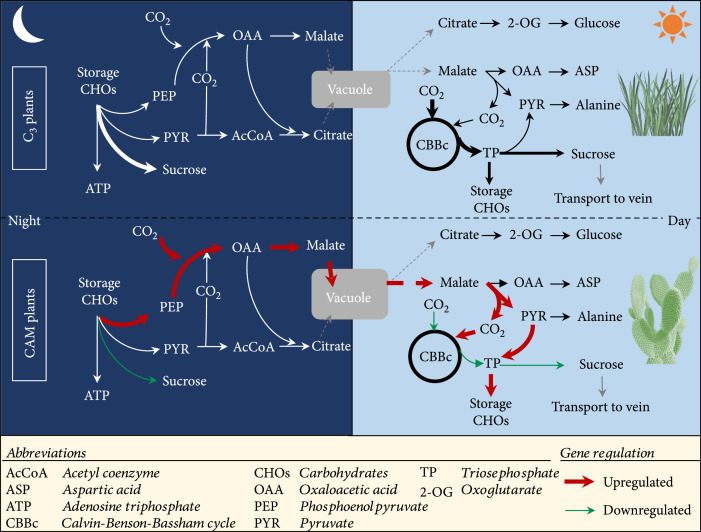
Daytime and nighttime metabolism of organic acids in C_3_ and CAM plants. Arrow thickness denotes flux. Adapted from [67].

**Figure 3:**
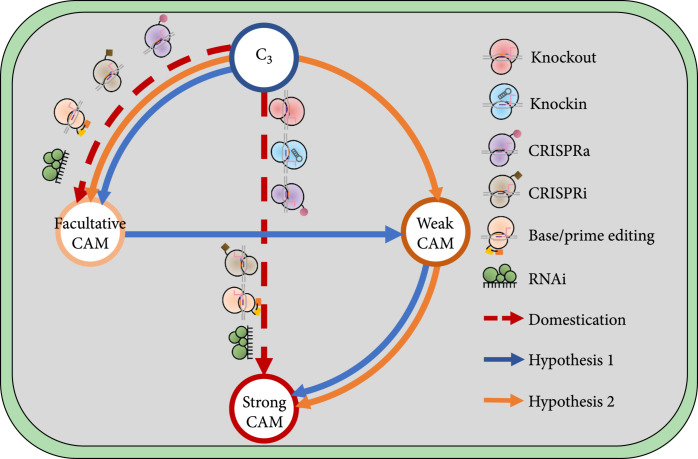
An evolution-based conceptual framework for crassulacean acid metabolism (CAM) engineering guidance. Hypothesis 1: CAM evolution followed a linear course leading from facultative CAM to strong constitutive CAM. Hypothesis 2: facultative and constitutive CAM evolved independently. The hypotheses were adapted from [18].

In different environments, ontogenies, and species, CAM-mediated CO_2_ fixation accounts for <1% to 100% of total carbon gain [72–74]. CAM plants may be facultative (i.e., reversible induction or upregulation of the CAM pathway by environmental stress) or obligatory (i.e., mature photosynthetic tissues always perform CAM photosynthesis as a result of a preprogrammed, irreversible developmental process) [72, 74]. In addition, strong CAM and weak CAM are also widely used to define CAM species, with strong CAM meaning that ~95% of carbon intake is through the CAM pathway [72]. A recent comparative analysis of key carbon fluxes between C_3_ and CAM pathways showed that C_3_ plants had metabolite fluxes similar to CAM fluxes [67] (Figure 2). More recently, two alternative models have been proposed to explain the evolution of the CAM pathway [18] (Figure 3). In hypothesis 1, C_3_ plants evolved forward to facultative CAM, weak CAM, and strong CAM in a linear manner. Under hypothesis 2, C_3_ plants evolved into facultative CAM and weak CAM independently, and then, weak CAM further evolved into strong CAM. The C_3_-to-CAM continuum might explain the reversible induction of CAM by environmental stress in facultative CAM plants [67]. These hypotheses are consistent with the view that the distribution of facultative CAM is wider among vascular plants than that reported previously [72]. However, the idea of a continuum must be tempered by the evident anatomical constraints placed on the evolutionary trajectories of CAM species reflected in the bimodal distributions of C_3_+CAM and CAM plants revealed by large-scale *δ*^13^C isotopic and leaf thickness surveys [75].

**Figure 4:**
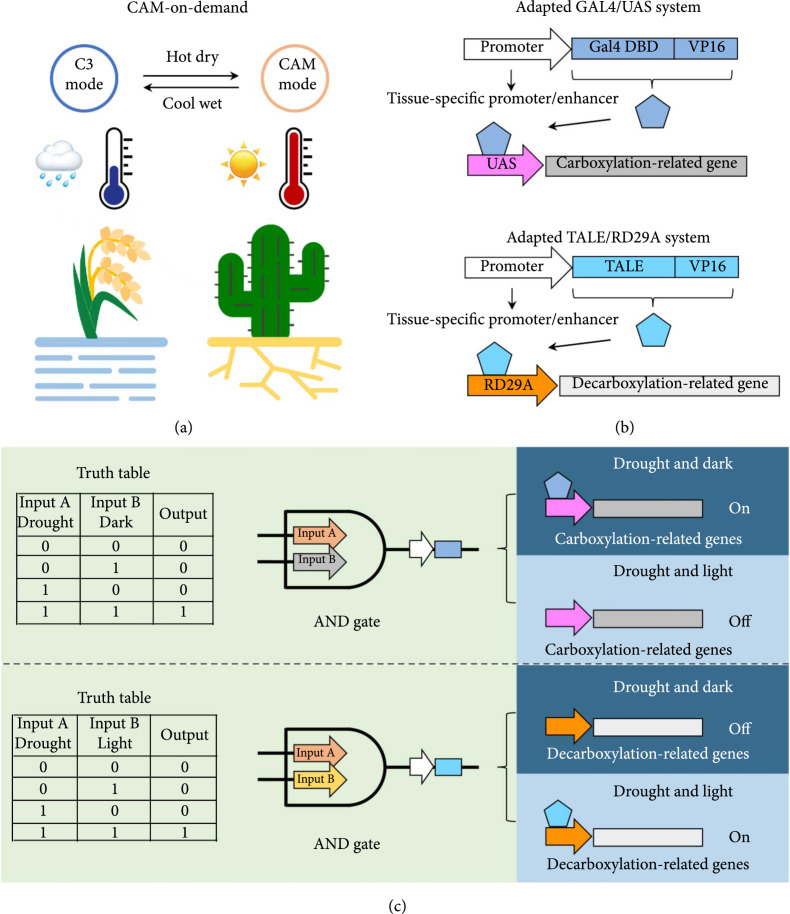
An inducible system for C_3_ crop in response to drought. (a) A CAM-on-demand system. (b) Sequence-specific transcriptional activation systems. (c) Boolean logic gates mediated CAM signaling systems. A value of 1 represents a true answer, and 0 represents a false answer.

Seasonal drought stress is a widely existing challenge for crop production, and this challenge could be potentially addressed through engineering a drought-inducible CAM or CAM-on-demand system [9, 30, 76]. In facultative CAM plants, CAM metabolism can be induced and reverted to the C_3_ mode multiple times by water deficit, salinity, and high light [18, 72, 74], implying that C_3_ photosynthesis can be engineered to be metabolically compatible with the water-use efficient adaptation. A typical CAM-on-demand system represents an engineered photosynthesis system that enables reversible CAM induction in response to drought stress (Figure 4(a)). In particular, CAM-on-demand plants would operate in the C_3_ mode under moisture and cool conditions and temporarily switch to the CAM mode if the environment turns hot and dry. Such a system could not only possess a feature of drought tolerance under the CAM mode, but also maintain a relatively high growth rate of biomass accumulation under the C_3_ mode, resulting in a promising strategy in response to climate change. Therefore, from a CAM evolution-informed point of view, we can infer the following principles for CAM engineering: (1) there is no need to transfer a large number of genes from CAM species into C_3_ species (it is possible that the C_3_-to-CAM transition can be achieved through rewiring of temporal gene expression and rechanneling of existing metabolic flux) and (2) CAM-on-demand systems can be engineered through reversible drought-induced gene expression.

## 4. Installation of CAM-on-Demand Systems Using Gene Editing and Synthetic Biology Approaches

### 4.1. Genome Editing and Gene Regulation Approaches Required for CAM Engineering

There is a major difference in gene expression between facultative CAM and C_3_ plants, with facultative CAM plants featuring drought-inducible expression of genes related to CAM [72]. Recently, rapid development of the CRISPR technology has provided a very powerful toolbox for basic and applied biological research. For example, the CRISPR/Cas systems can be used to generate single- or multinucleotide replacements, insertions, and deletions in the genome using CRISPR/Cas9, base editors, and prime editors and to manipulate gene expression using CRISPR activation (CRISPRa) and CRISPR interference (CRISPRi) [77–79]. These tools have been used across different species, including *E. coli*, yeast, human cells, and plants. CRISPRa and CRISPRi can be used for the transcriptional gene regulation to facilitate C_3_-to-CAM transition, where compatible CRISPRa and CRISPRi systems promise to minimize the scale and complexity of biosystems design and engineering (Figure 3). Recently, CRISPRai was developed to simultaneously activate and inhibit gene expression in mammalian cells [80]. Developing such a CRISPRai tool in plants will clearly facilitate CAM bioengineering.

Alternately, small RNA-based RNA interference (RNAi), which regulates gene expression at the transcriptional and posttranscriptional levels, can be used together with CRISPRa for simultaneous gene activation and inhibition in plants [81, 82]. Additionally, a new gene editing method termed prime editing can perform targeted small insertions, deletions, and base swapping in a precise manner in yeast and human cells [83]. Recently, prime editing was successfully applied in plant species such as rice and wheat, although the editing efficiency is much lower than that of the mature CRISPR editing tools [84, 85]. In short, prime editing, together with base editing, will be useful for creating genomic mutations, such as single-nucleotide change and multinucleotide mutations (replacement, insertion, or deletion), required for C_3_-to-CAM progression [78]. Lastly, CRISPR knockout and knockin can be used for CAM engineering. Considering that the number of CAM-related genes is high, multiplex genome editing and regulation will be needed to accelerate the discovery and functional characterization of these genes, as well as to facilitate the engineering of functional CAM-related genes in C_3_ species. CRISPR/Cas9-enabled multiplex knockout and CRISPRa are available in plant systems [86, 87]. Engineering CAM in C_3_ plants will require biosystems design approaches such as CRISPR-based multiplex gene editing and gene regulation, engineering of drought-responsive gene circuits, and rewiring of metabolism.

### 4.2. Establishing a Drought Stress Signaling Pathway

A distinguishing feature of the CAM-on-demand system is that it requires drought-inducible transcription of CAM-related genes. Specifically, CAM-on-demand will require the regulatory expression of carboxylation-related genes under drought and dark conditions, while the expression of decarboxylation-related genes will be needed under drought and light conditions. Identifying a sensor that is capable of reading multiple inputs and transmitting them to a downstream network will be indispensable. In a general context, Boolean logic gates mediate synthetic genetic circuits that can convert multiple input signals. Such circuits have been successfully implemented in various biological systems, such as yeast and mammalian cells [88, 89]. Boolean logic gates convert multiple input signals into “truth” values, where a value of 1 represents a true answer and 0 represents a false answer [90]. Typically, following a set of algorithms, these synthetic genetic circuits can generate a defined response through an integration of multiple molecular input signals [91]. A synthetic gene circuit based on an AND gate, which generates an output only when two input signals are present, can be used for drought-inducible expression of CAM-related genes. As illustrated in Figure 4(c), if inputs A and B are defined as drought and dark signals, respectively, then the downstream carboxylation-related genes cannot be activated unless both drought and dark conditions are met. In this case, drought-induced positive transcriptional regulators and dark-inducible switches for the regulation of gene expression can be integrated to define the inputs A and B. In the context of CAM, the family of abiotic stress-responsive transcription factors (TFs), including *NAC*, *bZIP*, *WRKY*, *NF-Y*, *MYB*, and *AP2/ERF*, has been identified and characterized [92]. Recently, a plant stress response system was engineered, which employed the receptor for the plant stress hormone ABA and chemical agonists for initiating a response to drought [93, 94]. In addition, in prokaryotic and eukaryotic systems, many optogenetic switches responsive to green, UV-B, blue, red, and far-red/near-infrared light have been developed and tested to control intracellular signaling pathways with a high spatial and temporal resolution [95]. Furthermore, several optogenetic systems have been applied in plants, such as a CarH-based green light-regulated expression system and a phytochrome-based red light-inducible expression system [96–98]. Light-inducible expression systems with a broad spectrum will be required to optimize a CAM-on-demand system where entrainment of circadian-regulated CAM gene expression patterns will likely be necessary.

### 4.3. Establishing Gene Activation Systems

Secondly, an activation system that can simultaneously manipulate multiple carboxylation- or decarboxylation-related genes is needed. During the night, the metabolic fluxes from stored carbohydrates towards malate, including the intermediates PEP and OAA, are increased, but the flux towards sucrose is decreased in CAM plants (Figure 2). To achieve this feature in a C_3_ plant, the key enzymes and transporters, such as *β*-CA, PEPC, PPCK, NAD(P)-MDH, tDT, and ALMT, will have to be transcriptionally activated to establish the carboxylation module. To date, gene activation can be accomplished with multiple tools (as noted above) in plants, such as by using strong promoter-mediated overexpression, CRISPRa, and a TALE-mediated mTALE-Act system [87]. Among these, multiplex CRISPR-Act2.0 and mTALE-Act, which can manipulate multiple genes simultaneously, appear appropriate for this task. However, neither of these systems can activate more than four genes simultaneously based on the current technology. Therefore, a highly multiplex activation system is required to meet the needs of a fully functional carboxylation or decarboxylation module. Very recently, we developed a *de novo* multiplex CRISPRa system that can simultaneously perturbate the expression of eight genes in *A. thaliana* (Yuan et al., unpublished data). Such a system is necessary to simplify the assembly of genetic parts and lower the complexity of the intact model.

The initial target of the gene activation system will be PEP. The synthesis of PEP is indispensable in a fully functional carboxylation module because it is the key substrate for nocturnal CO_2_ fixation mediated by PEPC, which converts PEP and bicarbonate to OAA (Figure 1). Unlike C_3_ plants, in which the hydrolytic route mainly degrades starch, typical CAM plants degrade starch to provide a substrate for PEP mainly through the phosphorolytic pathway [24, 61]. Conceptually, C_3_ plants would benefit from an engineered switch from hydrolytic to phosphorolytic starch breakdown. Specifically, during the day, the metabolic fluxes from accumulated malic acid in the vacuole towards PYR and subsequent storage carbohydrates are increased, but the flux towards sucrose or other soluble storage carbohydrates can be decreased in CAM plants (Figure 2). To achieve this feature in a C_3_ plant, the key enzymes and regulators, such as NAD(P)-ME, PPDK, PPDK-RP, PEPCK, and others, would have to be transcriptionally activated to establish the decarboxylation module. As discussed above, the multiplex CRISPRa system can also be applied to this task.

In addition to the multiplex CRISPRa system, these engineered systems could be accomplished through a sequence-specific transcriptional activation system (e.g., an adapted GAL4/UAS system or an adapted TALE/RD29A system). The GAL4/UAS system, which was originally developed for studying gene expression and function in *Drosophila* [99], has become one of the most useful systems for targeted gene expression across different species. For instance, *Potri.002G146400*-encoded PtrEPSP was identified as a transcriptional repressor using a GAL4/UAS-mediated protoplast transient expression system in *Populus* [100]. Inspired by this work, an adapted GAL4/UAS system could be used to control carboxylation-related gene expression. This system consists of two individual parts serving for targeting and activation (Figure 4(b)). A Gal4-DNA-binding domain is fused to the transactivator VP16 (GD-VP16) to generate a transcriptional activator that targets the UAS enhancer, and GD-VP16 is driven by a tissue-specific promoter/enhancer. The other component is the carboxylation-related gene driven by a UAS enhancer. Also, the expression of GD-VP16 is regulated by an AND gate (Figure 4(c)). That is, under defined drought and dark conditions, GD-VP16 is bound to the UAS enhancer, thereby activating carboxylation-related gene expression. To provide further precision in expression, the GAL4/UAS system has been further characterized to increase the dynamic range of the system [101]. Simultaneously, an alternate independent activation system can be used to manipulate the decarboxylation-related gene expression. Here, transcriptional activator-like effectors (TALEs), containing a modular DNA-binding domain, can be used to generate chimeric transcriptional activators or repressors. A chimeric TALE-SRDX repressor can be used to repress the transcription of the transgene *RD29A::LUC* and endogenous gene *RD29A* in *A. thaliana* [102]. Again, inspired by this work, an adapted TALE/RD29A system could be used to control decarboxylation-related gene expression (Figure 4(b)). Similar to the GAL4/UAS system, one component is a TALE-DNA-binding domain-fused transactivator VP16 (TALE-VP16) driven by a tissue-specific promoter/enhancer and the other component is the decarboxylation-related gene driven by a *RD29A* promoter. The expression of TALE-VP16 is regulated by an AND gate (Figure 4(c)).

## 5. Iterative Design-Build-Test-Learn (DBTL) Cycles of CAM Engineering

**Figure 5:**
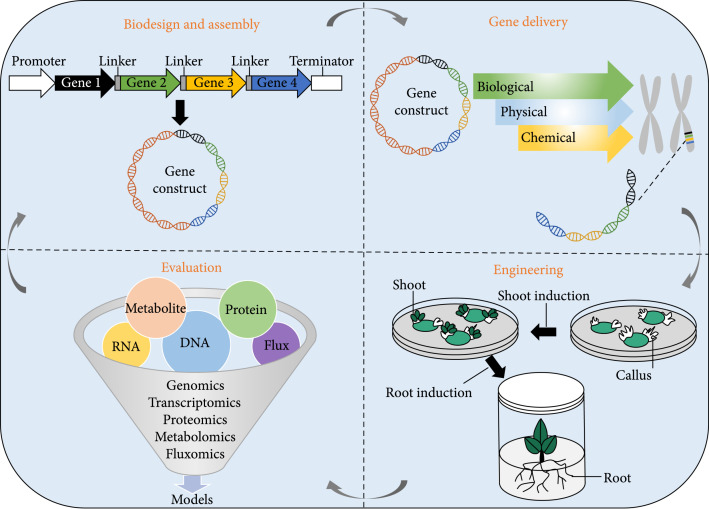
An overview of synthetic biology-dependent crassulacean acid metabolism (CAM) engineering.

The application of biosystems design to CAM engineering involves DBTL, which has four different phases: (I) biodesigned genetic circuits and assembly of multigene constructs, (II) delivery of biodesigned devices, (III) plant engineering, and (IV) evaluation of engineered plants (Figure 5). In phase I, synthetic devices will likely be essential components of CAM engineering. Although different synthetic switches and biosensors for controlling genome editing, gene regulation, and protein stability have already been utilized in plants, deployment of more complicated genetic circuits for genetic engineering in plants is still a big challenge [90]. This challenge is primarily caused by the experimental bottlenecks (e.g., lack of efficient plant transformation systems) and slow generation times of plants making it difficult to test the genetic circuits in plants. To overcome these limitations, protoplast-based and *Agrobacterium*-mediated leaf infiltration transient expression assays could be used to provide a rapid and robust analysis of transgene expression and protein subcellular localization and interaction [103]. For the assembly of multigene constructs, there are multiple methods of DNA assembly available, including Gibson assembly, BioBrick assembly, Golden Gate assembly, TOPO cloning, Gateway cloning, TNT cloning, and traditional restriction enzyme cloning [16, 104–106]. Among them, the Golden Gate assembly is capable of assembling up to 24 DNA fragments in a seamless and highly efficient manner [107]. However, unexpected interactions or transcriptional interferences between neighboring transcription units in multigene constructs are commonly found in all eukaryotic organisms including plants [108, 109]. To facilitate modular construction of a CAM gene circuit composed of multiple transcription units, which must have different diel expression patterns, transcriptional interference should be avoided. To overcome this issue, genetic insulators (enhancer blocking or barrier activity) could be deployed in multigene constructs that possibly prevent these unwanted interactions and increase transgene expression in plants [110, 111].

In phase II, the conventional methods to deliver genes to plant cells can be classified into three categories: biological, physical, or chemical approaches, with the most common and preferred method being *Agrobacterium*-mediated plant transformation. However, to insert large constructs containing multiple genes into the plant genome with high structural and functional stability of the engineered gene modules, new methods need to be developed for multiple rounds of site-specific *in planta* gene stacking [112]. In phase III, tissue culture-based plant transformation is widely used to create transformed or genome-edited plants. However, creating transgenic plants through tissue culture is a bottleneck of genome editing in plants, because (1) it is only suitable for a limited number of species and genotypes, (2) it is time-consuming with low efficiency, and (3) it might cause unwanted genetic and epigenetic changes [113]. To overcome this bottleneck, two methods were recently developed for the generation of gene-edited dicotyledonous plants via *de novo* meristem induction by developmental regulators without *in vitro* culture [114]. In phase IV, robotic high-throughput phenotyping [115], in combination with omics approaches, is needed to advance functional analysis for a quick evaluation of biodesigned devices and circuitry in the transgenic or genome-edited plants. The omics-based system dynamics modeling and diel flux balance analysis [23, 116, 117] will need to be performed for reconstructions of metabolic networks to improve the performance of transgenic plants engineered with CAM. In order to optimize the biosystems design for CAM engineering, multiple iterations of the DBTL cycle will be required and possible adjustments will be made to increase precision and efficiency in each iteration.

## 6. Conclusion and Perspectives

The engineering of CAM and coadaptive traits, such as tissue succulence, holds a great potential for sustainable production of fiber, food, feed, and biofuels in water-limited areas [9, 15, 19, 60]. Initially, a deep understanding of CAM-related gene function is a key prerequisite for engineering CAM into C_3_ crops [30]. Many such genes have been identified and organized into separate CAM-related modules (i.e., carboxylation, decarboxylation, and stomatal regulation). The minimum genes that are indispensable to maintain a functional module are proposed based on the knowledge of genomic research and comparative analysis. Genes that play important roles in the CAM pathway are summarized in Table 1, providing a database to guide the user in CAM engineering. Despite decades of notable progress in CAM research, a number of potentially important genes may be yet undiscovered. Comparative analysis of more CAM plant genomes will be needed to accelerate the identification of biological parts (e.g., enzymes, posttranslational modifiers, transporters, and transcription factors) for CAM engineering.

Additional research will be required to characterize the function of candidate genes inferred from the omics and comparative genomics research. Efforts will be needed to reduce the redundancy of CAM-related genes found in different CAM species. To accelerate the C_3_-to-CAM engineering, wiring of appropriate temporal gene expression and rechanneling of existing metabolic flux will be essential. A CAM-on-demand system that responds to episodic or seasonal drought can be achieved in C_3_ plants through reversible drought-induced gene expression to increase WUE. Considering the genes mentioned above, using single-cell technologies will enable exploration of photoperiod and cell-type dynamics of CAM-related modules. By integrating with single-cell transcriptome data, the CAM modules can establish the layer of CAM regulation, which is incomplete. The regulatory network of CAM for each module can be explored by various approaches that have not been adapted to CAM research, including ATAC-Seq (Assay for Transposase-Accessible Chromatin followed by high-throughput sequencing), DAP-Seq (DNA affinity purification and sequencing), and DNase-Seq (DNase I hypersensitive site sequencing), which may provide decondensation of accessible chromatin regions that enrich motifs of transcription factors (e.g., *NAC*, *bZIP*, *WRKY*, *NF-Y*, *MYB*, and *AP2/ERF*) mentioned above [118–120].

With the nexus of new technologies like systems genetics, genome editing, synthetic biology, and gene activation systems, we are on the threshold of purposefully accelerating C_3_-to-CAM progression. The CRISPR toolkit for genome editing and gene regulation provides useful tools required for CAM engineering. The engineering of synthetic circuitry in plant systems has the potential to advance our understanding and ability to manipulate genetic and metabolic networks such as CAM. The strategies for building CAM-on-demand systems are feasible using coordinated systems and synthetic biology. To achieve these goals, synthetic genetic circuits for signaling and tools for manipulating multiple gene expression simultaneously at a large scale need to be developed in plant systems. Meanwhile, some technical challenges need to be overcome. For example, the plant transformation with large-scale multigene stacking that ensures different CAM modules to be properly expressed in transgenic plants will remain a challenge for the foreseeable future. Regardless, with the effective and successful demonstrations already reported in different organisms, synthetic biosystems design holds a great promise to enable C_3_-to-CAM progression in the near future.
